# Internet communication with the Local Community about Safe Community values 

**Published:** 2019-08

**Authors:** Jovan Vignjevic, Mirjana Milankov, Miodrag Blizanac, Mila Radovanovic

**Affiliations:** ^*a*^National Center for Injury Prevention and Safety Promotion, Novi Sad, Serbia.; ^*b*^Nongovernmental organization Faith, Love, Hope, Novi Sad, Serbia.

## Abstract

A Safe Community is a concept that is still unknown to the vast majority of the citizens of today s population. We, as representatives of the Safe Community and advocates of healthy and safe habits, have the task to digest the opportunities and values that a Safe Community provides as well as the way of thinking of a safe community. A Safe Community is related to the local community and it is therefore very important to learn the right ways of online communication with the local community on topics related to a Safe Community and everything that this concept encompasses. We need to promote values that are the concept of the Safe Community. First and foremost, we need to define stakeholders, specifically the target group we are addressing when this topic is at stake. Based on this, we will determine models and ways of communication with specific target groups. After that, we need to define the goals we want to achieve through a promotional campaign. By defining goals, we will extract the values of the Safe Community that we want to present as a key in promoting this concept and way of life within a local community. The next step in developing an Internet Communication Strategy with the local community is to select communication channels. Today, it’s very popular social networks and large amounts of information are shared through these channels of communication. For this reason, it is a very important segment of creativity and how to send a message for the target group. The younger population likes to use Instagram or Snapchat, but while the rest of the world’s population uses social networks like Facebook, LinkedIn, Twitter and etc.... It’s a very important website. Content that promotes the values of the Safe Community on an official website can be promoted through Google tools like Google Ads (Google AdWords) by selecting certain keywords and phrases.

**Keywords:**

Communication, Safe Community, Online Communication, Internet Communication, Social Network, Communications

